# Personality and motivation of physical activity in adolescent girls: effects of perceived parental support and social physical anxiety

**DOI:** 10.1186/s12889-024-18060-5

**Published:** 2024-03-13

**Authors:** Zahra Fathirezaie, Georgian Badicu, Fatma Hilal Yagin, Mohamadtaghi Aghdasi, Seyed Hojjat Zamani Sani, Kosar Abbaspour, Özgür Mülazımoğlu Ballı, Sara Entezar, Luca Paolo Ardigò

**Affiliations:** 1 Motor Behavior Department, Physical Education and Sport Sciences Faculty; Brain & Movement research group, Research Center of Biosciences and Biotechnology (RCBB), University of Tabriz, 51666 Tabriz, Iran; 2 Department of Physical Education and Special Motricity, Faculty of Physical Education and Mountain Sports, Transilvania University of Braşov, 500068 Braşov, Romania; 3 Department of Biostatistics and Medical Informatics, Faculty of Medicine, Inonu University, 44280 Malatya, Türkiye; 4 School of Sport Sciences and Technology, Pamukkale University, Pamukkale, Türkiye; 5 Department of Teacher Education, NLA Høgskolen, Linstows gate 3, 0166 Oslo, Norway

**Keywords:** Narcissism, Motivation, Physical activity, social physical anxiety, parental support

## Abstract

In the present study, we investigated the relationship between personality and motivation for physical activity while introducing perceived parental support and social physical anxiety in adolescent girls (*N* = 318, M_age_: 16.19 ± 0.51 years). The present study was a retrospective correlational study that was conducted to analyze of a path model. Dark triad traits: Machiavellianism, narcissism, and psychopathy, student’s motivation for physical activity, social physique anxiety, and participants’ perceptions of parents’ behaviors were measured. The findings indicated that psychopathy and Machiavellianism were directly and indirectly associated with motivation for physical activity, but Narcissism could only directly predict the motivation for physical activity. Also, need-thwarting (the most), need-supportive and social physical anxiety could predict motivation for physical activity. This model of the result suggests that among adolescent girls, dark triad personality could, directly and indirectly, predict motivation with need-supportive and need-thwarting and also social physical anxiety. It seems that the sense of importance and more attention to oneself in adolescent girls, which exists in the narcissistic personality, can directly lead to more motivation for physical activity. Also, the duplicitous ways of Machiavellian people in pursuing their motives were confirmed in this research.

## Background

Over the span of years, extensive research on dark personality traits [1]. Most of the studies on “dark triad personality” has centered on three traits that are commonly depicted: Machiavellianism, narcissism, and psychopath [2]. These characteristics capture individual differences in manipulativeness, cynicism (i.e., Machiavellianism), grandiosity, exhibitionism, superiority (i.e., narcissism), interpersonal antagonism, and callousness (i.e., psychopathy). Cold and manipulativeness, centering on personal interest, being pessimistic, and ignoring morality are features of Machiavellianism. Narcissism is known by these attributes: selfishness, weak empathy, the requirement for admiration, and supremacy. At last psychopathy refers to some anti-social behaviors, impaired sympathy, deficient psychological reactions, looking for excitement, and regret in the behavior [2–4]. In addition to genetics, the dark triad of personality is also influenced by environmental factors [4]. One environmental condition that may impact the improvement of the dark triad traits may be perceived parental support.

In addition to genetic factors, environmental factors such as parenting, counting autonomy support, and control have essential effects on personality characteristics. To foster autonomy, one must consider issues from the kid’s perspective and provide opportunities for the child to feel free to choose and act independently [5]. Parental control, on the other hand, refers to forced and intrusive parenting, which results from parents failing to consider matters from their children’s perspectives or pressuring them to satisfy their desires [6]. Parenting style and the dark triad features are linked by the self-determination theory (SDT) [7, 8] and life history theory [9–11]. The relationship between SDT and motivation for physical activity can be understood through the lens of the theory’s basic psychological needs. In the SDT, the need for autonomy refers to the sense of volition and choice in one’s actions. Additionally, the need for competence necessitates opportunities for gradually escalating levels of challenge within an activity to foster the development of higher levels of mastery. Finally, the need for relatedness pertains to the necessity for a compassionate and supportive social network characterized by robust interpersonal connections. When individuals feel that they have control over their exercise choices and that their physical activity aligns with their values, they are more likely to experience intrinsic motivation and sustained engagement in physical activity. Also, when individuals engage in physical activity in a supportive and social environment, their motivation for exercise is bolstered by positive social interactions and a sense of community [7, 8].

The main problem with life history theory is the distribution of bioenergetic and material resources by humans and other species [9–11]. According to research by Chen et al. [9] and Dunkel et al. [12], parenting practices have an impact on how children construct their life history strategy. According to SDT, a setting that promotes autonomy helps people express their personalities [8]. According to parental traits, parental autonomy support [5] tends to create a stable and safe environment for children; in contrast, parental control creates an unstable and insecure environment [8]. More specifically, children who grow up in an unstable and uncertain environment (such as one where resources vary) that their parents help create are more likely to adopt a fast life history strategy, which emphasizes the desire for instant gratification and leads to offensive, hasty, and impulsive behaviors; in contrast, children who grow up in a stable and predictable environment prefer to pursue a slow life history strategy, which promotes the desire for long-term benefits [9, 10, 13].

Several experimental studies have explored the potential of parenting to foresee children’s characters, counting the dark triad traits and other personalities [14–16]. Additionally, the findings of review studies show that individuals with high levels of psychopathy perceive their early family environments as being marked by parental rejection, neglection, or detachment, coupled with inconsistent or severe reprimand, and lacking in nurturing [17–19].

In addition, Machiavellianism and psychopathy were adversely correlated with adolescents’ views of parental relational support [3]. According to some psychologists [20], parents who give their kids excessive satisfaction and indulge them excessively are to blame for higher levels of narcissism in adults. Additionally, some findings suggest that the interaction of a demanding environment and negligent parenting (i.e., roughness and neglect) can promote an opportunistic interpersonal style, which is a common characteristic of people with high levels of Machiavellianism [21]. In addition, there is evidence that need thwarting behaviors by parents are associated with negative outcomes for their children. For example, a study by Bach et al. [22] found that a father’s need thwarting behaviors can lead to an increased risk of emotional disorders among their children. Moreover, researchers have shown that harsh parental practices can lead to the development of personality disorders among their children, potentially increasing the likelihood of subsequent negative outcomes such as substance abuse and criminal behavior.

It is given that the dark triad trait is related to anxiety [23]. Miller et al. [24] contended that there were correlations between the dark triad and some mental states, such as anxiety. Concerning the relationship between the dark triad and anxiety, results are blended and uncertain; for this case, there is reason to believe that, among individuals scoring high on the dark triad traits, higher scores could also be expected for anxiety [25]. In contrast, there is also developing back for a connection between the dark triad and low anxiety [26]. Anxiety disorders are the foremost common disorders in adolescents [27], one of these disorders is social physical anxiety (SPA). It refers to a sense of confusion or fear about others assessing one’s bodily form in a negative way [28]. Actually, physical changes related to puberty and the expanded importance of peer assessments and acceptance driving up to and during adolescence can increase one’s feelings of social awareness and self-consciousness [29]. Thoughts and feelings about one’s body, make SPA an issue for both mental and physical health results [29] it means that social physique anxiety could be derived from a perceived disagreement between the perfect and existent body image [30]. So, it has been concluded that people who have a negative idea about their bodies are more susceptible to experiencing SPA, and they might be avoiding self-presenting situations in sports and exercise [31].

The main focus of personality research in sports psychology was to look at the relationships between participation, personality, and athletic success [32]. It has been shown, that personality traits have developed as one way to better understand who may be at risk for low exercise participation [33, 34]. Factors that could improve the physical activity intervention achievements and health results have received little study; but some proof from observational studies [35] recommends that personality clarifies some of the natural variations in physical activity. For instance, all three of the dark triad traits are driven by ambition and power, and these traits are typically linked to external, instrumental drives [36]. Narcissistic people are extremely motivated. This is in line with the findings of a prior study, which found that a higher level of narcissism was associated with both internal and external incentives [37]. Individuals with high levels of Machiavellianism and psychopathy, however, will not be driven by internal causes [37]. Some studies suggest that personality traits apply their effect on social cognitions such as physical activity attitudes, and norms perception [35]. Recent research on the effective ways to promote PA in childhood showed that the well-designed intervention based on SDT-informed need-supportive training for parents, enables them to interact with their children to support their intrinsic motivation towards leisure-time physical activity [38]. In addition to these issues, factors such as personal problems, social beliefs, government thought, cultural attitudes, legal- law and customary barriers, were identified as the central issue of social cultural barriers of Muslim women athletes to participate in sport.

In this regard and the following conceptual models show the relationship between parents’ behaviors and personality traits [39], this study aimed to measure the effect of variables on PA motivation. For this purpose, the direct and indirect relations of dark personality traits (Machiavellianism, Psychopathy, and Narcissism), participants’ perceptions of parents’ behaviors (need-supportive and need-thwarting), and social physical anxiety on PA motivation were investigated to identify the strongest factors as well as the effective pathways in the model. Therefore, two basic hypotheses were examined in this research: the first hypothesis stated that there is a direct relationship between personality traits and physical activity motivation. And the second hypothesis stated that there is an indirect relationship between personality traits taking into account the factors of parental behavior and social physical anxiety with physical activity motivations.

## Methods

### Participants

G*Power software version 3.1.9.4 was used to estimate the sample size. The number of predictors in our study was equal to 6 and the effect size was estimated to be 0.1, the alpha error probability was 0.05, and the power of the test was 0.95. Although according to the calculations, the number of samples equal to 215 people was sufficient, however, due to online data collection and daily control of the participation rate, 318 people who had attended virtual physical education classes (age: 16.19 ± 0.51 years) participated in the research from 9 schools in Tabriz City, Iran. They were selected by cluster sampling and voluntarily participated in this study. The criteria for entering the research included the following, which were collected by self-report: Being 15 to 18 years old, the absence of motor and mental problems, long-term insomnia, and illness, and participation in sport or physical activity.

Also, the possible economic situation (according to the collection of data from an urban area at an above-average level) was controlled. Sports history and type of sport, parents’ education level, height (M = 1.63 ± 0.60 m) and weight (M = 56.72 ± 11.32 kg) were measured.

To investigate the motivation to participate in physical activity, the motivation to participate in virtual physical activity classes held by schools during the coronavirus pandemic was considered. These classes were adapted to the conditions with an emphasis on physical fitness factors in a regular and planned number of school sessions, including balance, strength, muscular endurance, cardiorespiratory endurance (aerobic exercises) and neuromuscular coordination.

### Procedure

The present study was a retrospective correlational study that was conducted to make an applied model. Participants completed the following measures and demographic characteristics through an online questionnaire. Participants completed the questionnaires at the end of the semester. After notifying the student’s parents and signing the informed consent form, participants filled out a booklet of self-rating including dark triad, motivation for physical activity, social physique anxiety, interpersonal behaviors questionnaires, and sociodemographic information. This study was performed consistent with the seventh edition of the Helsinki Declaration and the Sports Sciences Research Institute of Iran’s local ethics committee approved the study.

## Measures

### Dark Triad personality

Three personality traits known as Machiavellianism (sample item: I tend to manipulate others to get my way), psychopathy (sample item: I tend to lack remorse), and narcissism (sample item: I tend to want others to admire me) were measured with the Dark Triad Dirty Dozen [26]. Each personality trait is measured by self-report with 4 items on a 7-point Likert scale (1 = strongly disagree; 7 = strongly agree). The psychometric properties of the Persian version of the questionnaire have been already confirmed [40, 41] (Cronbach’s alphas between 0.74 and 0.85). The internal consistency in the present sample was reasonable (Cronbach’s alpha = 0.68).

### Sports participation motivation questionnaire

Sports participation motivation questionnaire (PMQ) is a 30-item scale (sample item: I am physically active because… I want to play sport at a higher level) consisting of 8 sub-dimensions (achievement/status, physical fitness/working off energy, team membership/spirit, friendship, fun, competition, skill development, and motion/being active) [42]. The reliability of this questionnaire was reported by Zahariadis and Biddle [43] using Cronbach’s alpha coefficient as 0.80. In the present study, internal consistency coefficient of the scale was calculated as 0.86.

### Social physique anxiety

The Social Physique Anxiety Scale (SPAS) with 12 items (sample item: I am comfortable with the appearance of my physique or figure) was used to check out anxiety of situation-specific concerning the social judgment of one’s body during the sessions of physical education. Students responded to items on a scale from not at all characteristic of me = 1, to extremely characteristic of me = 5 [28]. The internal consistency of the SPAS was reported to be 0.90, and the 8-week test–retest reliability was 0.82 [44, 45] and also in adolescents [46]. The Cronbach’s alpha in the current sample was 0.73.

### Interpersonal behaviors questionnaire

A modified 24 items version of the Interpersonal Behaviors Questionnaire (IBQ) [47] by adding the stem ‘My parent…, was used to assess participants’ perceptions of parents’ need-supportive (sample item: Gives me the freedom to make my own choices) and need-thwarting (sample item: Pressures me to do things their way) behaviors. Competence, autonomy, and relatedness support and thwarting are the factors of need-supportive and need-thwarting each of which is measured by 4 items on a 7-point Likert scale (1 = not to all true; 7 = very true). The confirmatory factor analysis and fit indices of the Persian version of this scale confirmed the six-factor model with the three need-supportive and the three need-thwarting constructs [47]. The reliability (Cronbach’s alpha) of this questionnaire in the present study was 0.71.

### Statistical analysis

First, we calculated descriptive statistics. Then Pearson’s correlation coefficients were used to examine the association between the variables, which gave a piece of knowledge into how the different variables may affect one another and helped to detect an initial model. Based on values of skewness and kurtosis all variables were ordinarily distributed (range of < − 2, 2>). The linearity assumption is checked by inspection of scatterplots to be sure that pairs of variables were chosen randomly. Finally, we used path analysis to demonstrate the relation between dark personality traits and motivation for physical activity with a moderating effect of parental behavior and social physical anxiety. We applied regression imputation to replace the missing value. Statistical Package for the Social Sciences 20.0 (IBM Corporation, Armonk, NY, USA) and LISREL 8.5 for Windows (Scientific Software International, Inc., Skokie, IL, USA) were used for the descriptive and correlational analyses and path analyses, respectively.

## Results

### Descriptive statistics and bivariate correlations between the main study variables

Table 1 shows that Narcissism was the most prevalent among the subjects.

**Table 1:** Descriptive statistics and zero-order correlations between the study variables

Variables	1	2	3	4	5	6	M	SD	Skewness	Kurtosis
1. Machiavellianism							6.37	2.57	1.07	0.53
2. Narcissism	0.12^*^						14.57	3.09	0.58	− 0.01
3. Psychopathy	0.37^**^	− 0.02					8.74	2.98	0.63	0.37
4. Need-thwarting	0.32^**^	0.00	0.30^**^				22.96	10.90	1.23	1.03
5. Need-supportive	− 0.24^**^	0.09	− 0.32^**^	− 0.71^**^			49.42	10.75	-1.29	1.35
6. SPA	0.16^**^	0.05	0.18^**^	0.29^**^	− 0.33^**^		28.41	9.93	1.03	0.68
7. Motivation	− 0.21^**^	0.21^**^	− 0.28^**^	− 0.16^**^	0.30^**^	− 0.16^**^	21.70	1.93	-1.08	0.38

Notes: * *p* ≤ 0.05; ** *p* ≤ 0.01.SPA: Social physical anxiety

Significant correlations were found among dark traits (Machiavellianism and Psychopathy), interpersonal behavior items, and social physical anxiety. However, there was no relationship between narcissism and interpersonal behavior items. Also, a close association was observed between motivation and dark traits which were positive between personality traits and narcissism and negative among personality traits, Machiavellianism and psychopathy. Moreover, there is also a significant relationship between interpersonal behavior items, Social physical anxiety, and motivation.

Based on *p* values, variables that did not have a significant relationship were not examined in the path analysis phase.

Consistent with the hypothesis, Machiavellianism, psychopathy, need-thwarting, and social physical anxiety with motivation were negatively correlated while narcissism and need-supportive positive relationship was found with motivation. There would be a negative relationship between SPA and need-supportive while a positive relationship between SPA and Machiavellianism, Psychopathy, and need-thwarting.

### Path analysis

Machiavellianism, narcissism, and psychopathy were all listed as three factors in the suggested model that were both directly and indirectly associated with motivation. We tested the initial path model as follows: dark personality traits were hypothesized to be related to all variables, while additionally, all three dark traits can predict the motivation to participate in physical activity through perceived parental support. It was expected that there would be significant relationships between perceived parental support and motivation, while dark traits would predict motivation through perceived parental support and social physical anxiety. In the initial model, seven direct paths from Machiavellianism to SPA (γ = 0.05, *t* = 0.8), psychopathy to need-thwarting (γ = 0.03, *t =* 0.8), narcissism to SPA (γ = 0.02, t = 0.32), narcissism to need-thwarting (γ = 0.04, t = 1.12), psychopathy to SPA (β = 0.07, *t =* 1.21), narcissism to need-supportive (γ = 0.1, t = 1.95) and from need-thwarting to SPA (β = 0.09, *t =* 1.11) were nonsignificant. This model could be improved upon, according to analysis, to better fit the data. To find a more frugal model, we deleted the seven nonsignificant pathways.

Finally, fit indices showed that the final model had a good fit to our data, including: Root Mean Square Error of approximation = 0.04 (reference value ≤ 0.10); CFI = 0.99 (reference value ≥ 0.95); NFI = 0.98 (reference value ≥ 0.95) and GFI = 0.99 (reference value ≥ 0.95). So, 20% of the motivation (Y_4)_ variance was accounted for by the variance of dark triad personality (x_1_, x_2_, x_3_), perceptions of parent’s need-supportive (y_1_) and need-thwarting (y_2_) behaviors and social physical anxiety (y_3_).

$${{\rm{Y}}_4} = -.14{{\rm{x}}_1} + -.16{{\rm{x}}_2} +.2{{\rm{x}}_3} +.29{{\rm{y}}_1} +.15{{\rm{y}}_2} + -.10{{\rm{y}}_3}$$

Contrary to expectations, the personality type of Machiavellianism (γ=-0.14) and psychopathy (γ=-0.16) was able to predict the motivation to participate in physical activity by modulating perceptions of parents’ need-supportive and need-thwarting behaviors and social physical anxiety. However, the personality types of Narcissism could not indirectly (through perceptions of parents’ need-supportive and need-thwarting behaviors and social physical anxiety) predict motivation. Narcissism could predict directly motivation (γ = 0.20).

**Fig. 1:**
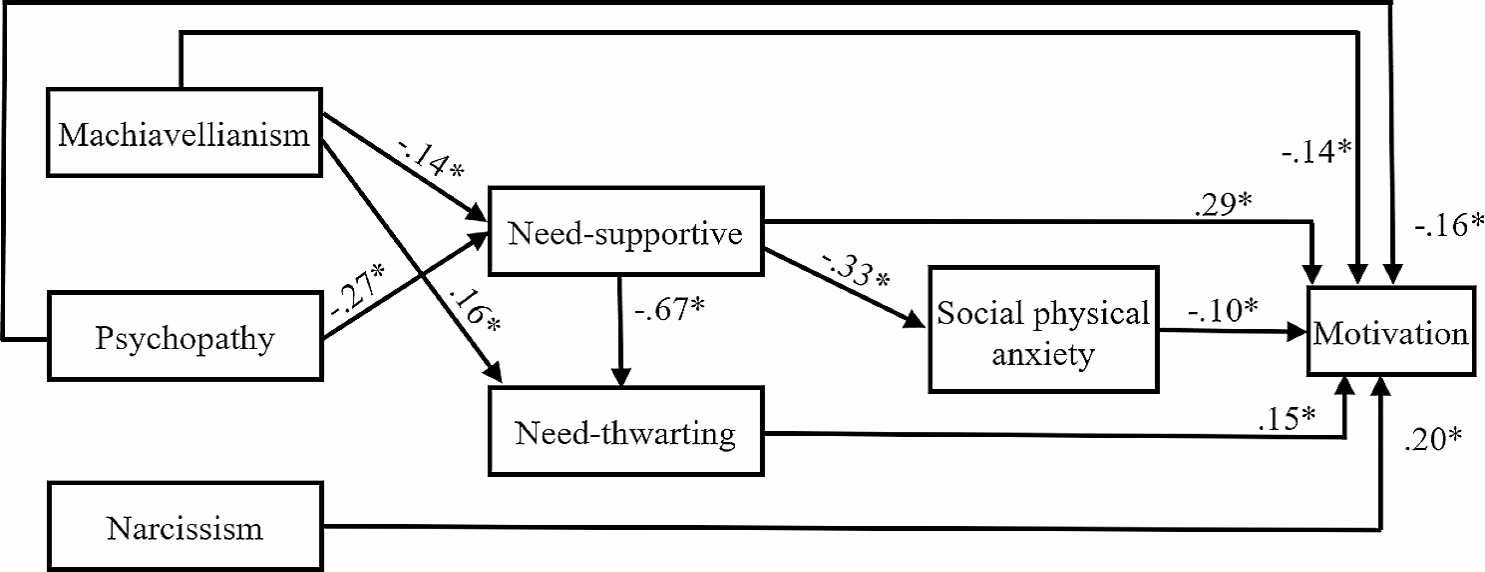
Standards path coefficients of the final model; *: *p* ≤ 0.05

In addition, as seen in Table 2; Fig. 1 need-supportive was able to directly predict motivation (β = 0.29) need-thwarting also directly predicted motivation (β = 0.15), and SPA was able to directly predict motivation (β= − 0.10).

**Table 2:** Direct, indirect, and total effect of variables in the model

	Direct	Indirect	Total
On Need-supportive			
Of Machiavellianism	− 0.14		− 0.14
Of Psychopathy	− 0.27		− 0.27
On Need-thwarting			
Of Machiavellianism	0.16	0.09	0.25
Of Need-supportive	− 0.67		− 0.67
On SPA			
Of Need-supportive	− 0.33		− 0.33
On Motivation			
Of Machiavellianism	− 0.144	− 0.006	− 0.15
Of Psychopathy	− 0.16	− 0.06	− 0.22
Of Narcissism	0.20		0.20
Of Need-supportive	0.29	− 0.08	0.21
Of Need-thwarting	0.15		0.15
Of SPA	− 0.10		− 0.10

SPA: Social physical anxiety

## Discussion

The goal of the present study was to determine the effect of the dark triad traits on motivation to participate in physical activities, under the influence of perceived parental support and social physical anxiety. This investigation focused on whether each factor could predict motivation positively or negatively. Also, the present study created a pathway where all three variables (Machiavellianism, Narcissism, and Psychopathy) were directly or indirectly related to motivation. These three personality traits are often referred to as the “Dark Triad,” and are characterized by a lack of empathy, manipulative behavior, and a focus on self-interest. Understanding the relationship between these traits and motivation is crucial for developing effective interventions and treatments for individuals who exhibit these traits.

According to our results, Narcissism could predict motivation positively. This supports Prusik and Szulawski’s investigation that Narcissists are motivated people [37]. Narcissism is characterized by a grandiose sense of self-importance, a need for excessive admiration, and a lack of empathy for others. Individuals with narcissistic traits are often motivated by a desire for power, prestige, and attention. They seek out opportunities to be admired and validated, and are often willing to manipulate others to achieve their goals. Research has shown that narcissists are more likely to engage in risky and aggressive behaviors in order to maintain their sense of superiority and control over others [48].

In addition, results from psychopathy and Machiavellianism showed a negative prediction on motivation, which proves that individuals high in psychopathy and Machiavellianism will not be motivated by internal factors [37]. The present results were also consistent with Muris et al. [3] that adolescents’ perceptions of parental relational support negatively related to Machiavellianism and Psychopathy.

The motivation behind these dark triad traits is complex and multifaceted. Research has shown that individuals with these traits are often driven by a combination of internal and external factors. Internal factors such as a need for validation, power, and control play a significant role in motivating individuals with dark triad traits. External factors such as social and environmental influences also contribute to the development and expression of these traits. For example, individuals who grow up in environments that reward aggressive and manipulative behavior are more likely to develop dark triad traits [26].

Furthermore, the findings support the fact that personality traits apply their effect on social cognitions such as physical activity attitudes [35], which in this case is the motivation to be physically active. However, our results were partially inconsistent with other researches. Sabouri et all [25] and Jonason and Webster’s [26] study showed the relationship between the dark triad and anxiety. This issue can be due to the type of variables. In the current research, social physical anxiety was considered, while in their research, anxiety was investigated. Moreover, the negative effect of SPA on motivation for physical activity in the present study was consistent with Ataly [31] that people who were more prone to experience SPA might be avoiding exercise because of self-presentation.

The lack of a relationship between narcissists and their parents’ need-supportive conduct contradicts recent claim that the adult with higher narcissism was actually a spoilt child who had received excessive indulgence from their parents [20].

Understanding the motivation behind these dark triad traits is crucial for developing effective interventions and treatments. Research has shown that individuals with dark triad traits are less likely to respond to traditional forms of therapy and are more likely to engage in manipulative and deceitful behavior during treatment. Therefore, interventions that focus on addressing the underlying motivations of these traits are more likely to be effective. For example, interventions that target the individual’s need for validation and control may be more successful in reducing the expression of dark triad traits [49].

As predicted, perceived parenting support is correlated to the motivation for physical activity. Our path model showed that need-supportive and need-thwarting positively predicted motivation. Perceived parenting support specific to physical activity did emerge as a positive predictor of adolescent motivation in physical activity in this sample. These results are inconsistent with the study of Saunders et al. [50] in which they showed a negative relationship between authoritative parenting and walking/cycling trips. The negative correlation between indulgent parenting and weekly walking/cycling trips may be explained by motorized transport support by parents, which itself reduces the need for children for active transportation.

Research has shown that the need for support and thwarting from parents can significantly impact an individual’s motivation for physical activity. According to SDT, individuals are motivated to engage in physical activity when their basic psychological needs for autonomy, competence, and relatedness are met. When parents provide support for their children’s physical activities, such as encouragement, praise, and involvement, it can enhance their motivation to participate in physical activities. On the other hand, when parents thwart their children’s need for autonomy and competence by being controlling or critical, it can decrease their motivation for physical activity [51].

A meta-analysis by Hancox et al. [52] also concluded that parental support positively predicted children’s physical activity levels, while parental thwarting negatively predicted physical activity.

In conclusion, perceived parental behavior, specifically the provision of support and thwarting, can significantly impact an individual’s motivation for physical activity. Parents play a crucial role in fostering their children’s motivation for physical activity by providing support and avoiding thwarting behaviors. Understanding the influence of parental behavior on motivation for physical activity can inform interventions and programs aimed at promoting physical activity in children and adolescents.

As shown in previous research, parenting behaviors were related to dark personality traits [14, 15, 39], however, this relationship was only observed in the Machiavellianism and psychopathy personality traits. The parenting effects are an essential feature of adolescent’s physical activity because they encourage children to the entrance and continuing of their involvement in any kind of physical activity, and help them to manage educational and social demands [53, 54]. Côté and Hay [54] cited that parents have an essential role in children’s life experiences and physical activity participation. Mizoguchi et al. [55] also showed a relationship between the father’s negative aspects influencing, and motivation among junior baseball players. It is shown that authoritative parenting and some family functioning (such as communication and affective involvement) are correlated with high physical activity among adolescents [56]. These results suggest that for adolescents, parenting support may be critical and influential to physical activity.

In line with our findings, the results of Fredricks et al. [57] approved that home experiences make children’s motivation for physical activity. According to their results, buying sports equipment by mothers related to higher ability and beliefs about sports among children. It seems that these purchases remind children of the importance of sports in life.

In addition, the results showed that social-physical anxiety can directly and negatively predict the motivation to participate in physical activity. Physical activity settings such as sports, exercise, and physical education are judgmental in nature, and stress is placed on the body’s form and ability. Also, these activities develop kinds of negative and positive emotional experiences [28]. Given these operationalizations, it is not surprising that social-physical anxiety experiences have been related to exercise involvement motivations, views, priorities, self-perceptions, and participation in or detachment of exercise. Social physical anxiety is a construct that refers to the fear of negative evaluation by others during physical activity. It is a common barrier to physical activity, especially among children and adolescents. Studies have shown that social physical anxiety can lead to lower levels of physical activity and poorer health outcomes [28].

Based on the results, practical implications were proposed as follows: The findings suggest that interventions targeting individuals with dark triad traits should focus on addressing the underlying motivations associated with these traits. Interventions that target the individual’s need for validation and control may be more successful in reducing the expression of dark triad traits. Also, this study highlights the duplicitous ways in which Machiavellianism can influence physical activity motivations. This suggests that interventions targeting individuals with Machiavellian traits should consider the complex nature of their motivations and behaviors.

The study highlights that parents play a crucial role in fostering their children’s motivation for physical activity by providing support and avoiding thwarting behaviors. Therefore, interventions and programs aimed at promoting physical activity in children and adolescents should consider the role of parental support.

Also, the study emphasizes the negative impact of social physical anxiety on motivation for physical activity. This suggests that efforts to promote physical activity should address the fear of negative evaluation by others during physical activity, especially among children and adolescents. Strategies to reduce social physical anxiety can contribute to increased participation in physical activities. The study suggests that interventions aimed at increasing parents’ need-supportive behavior to enhance their child’s motivation and physical activity could be beneficial.

Although the current research introduced a conceptual model and analysed that, however, the cross-sectional and retrospective nature of the study and the use of the self-report method is one of its limitations in generalizing the findings. Also, while physical activity motivation was assessed, objective physical activity needs to be assessed. In addition, future research could aim to increase parents need-supportive behaviour to increase their child’s motivation and physical activity. Specifically, future research could focus on developing specific classification systems of motivational behaviors to tailor interventions more effectively. For example, in a recent study by Ahmadi et al. [58], a classification system of motivational and behaviour change techniques was proposed that could be highly useful in future studies.

## Conclusion

Among the personality traits, Machiavellianism and psychopathy had a strong and significant relationship with need-thwarting and need-supportive behaviors, social physical activity, and motivation for physical anxiety. However, it was shown in the model that narcissistic syndrome has the greatest effect on physical activity motivation. However, two other traits (Machiavellianism and psychopathy) showed both direct and indirect effects on physical activity motivation. Two personality traits (Machiavellianism and psychopathy) were effective on need-supportive behaviors, and need-thwarting was only influenced by Machiavellianism. Therefore, it seems that Machiavellianism trait can influence physical activity motivations in duplicitous ways that are in the essence of this personality.

## Data Availability

The data presented in this study are available on request from the corresponding author. The data are not publicly available due to privacy and ethical restrictions.
